# Supplementary material for: Integrating genomic and multiomic data for Angelica sinensis provides insights into the evolution and biosynthesis of pharmaceutically bioactive compounds
Source: Commun Biol. 2023 Nov 24;6:1198. doi: 10.1038/s42003-023-05569-5 (PMC10674023; doi:10.1038/s42003-023-05569-5)
Supplement: Supplementary file 2 — Supplementary Information [file 42003_2023_5569_MOESM2_ESM.pdf]

Supplementary Figures and Tables

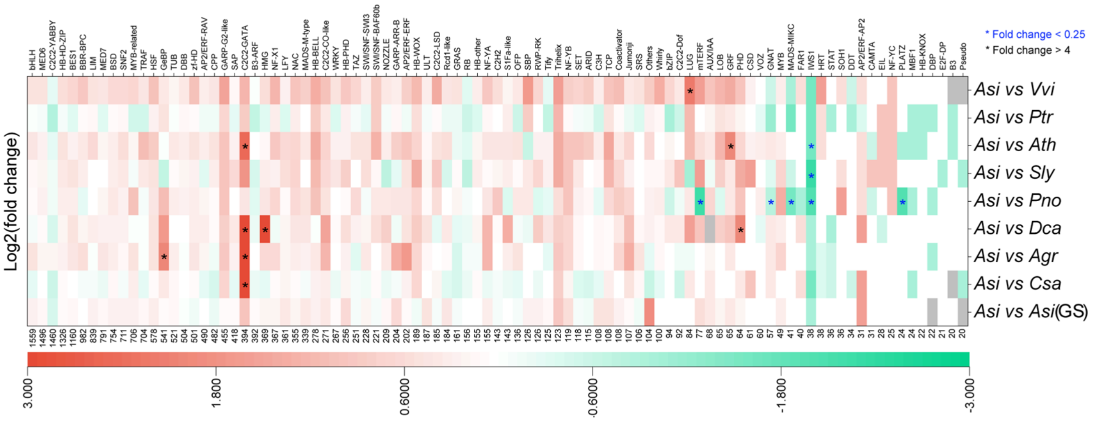

Figure S1. The heatmap constructed by the difference of transcription factor (TF) numbers between *A. sinensis* (QH) and other 8 species. The TF gene numbers of *A. sinensis* were displayed. There were more gene numbers of TF types, such as GeBP, HSF, GARP-G2-like, C2C2-GATA, C2C2-Co-like, HB-WOX relative to other species, while the number of C2C2-YABBY, B3-ARF and GRAS TFs decreased dramatically in *A. sinensis* compared with that in other species.

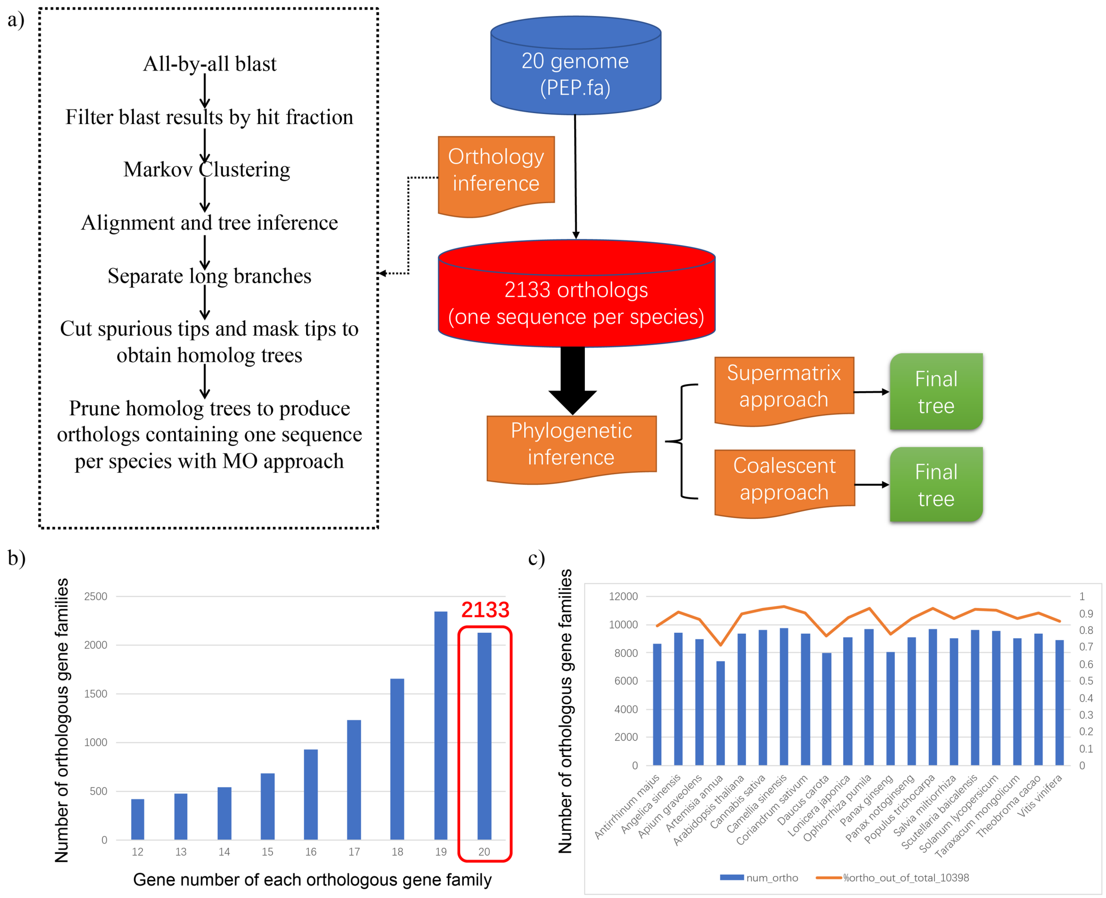

Figure S2. Overall workflow of construction of phylogenetic tree of 20 representative species.

a) Overall workflow of phylogenetic construction. We used a clustering and phylogenetic-based ortholog inference method, and Python scripts were obtained from https://bitbucket.org/yangya/phylogenomic_dataset_construction.

b) Gene number of each orthologous gene families. The 2133 orthologous gene families contain all 20 species.

c) Number of ortholog genes for each species.

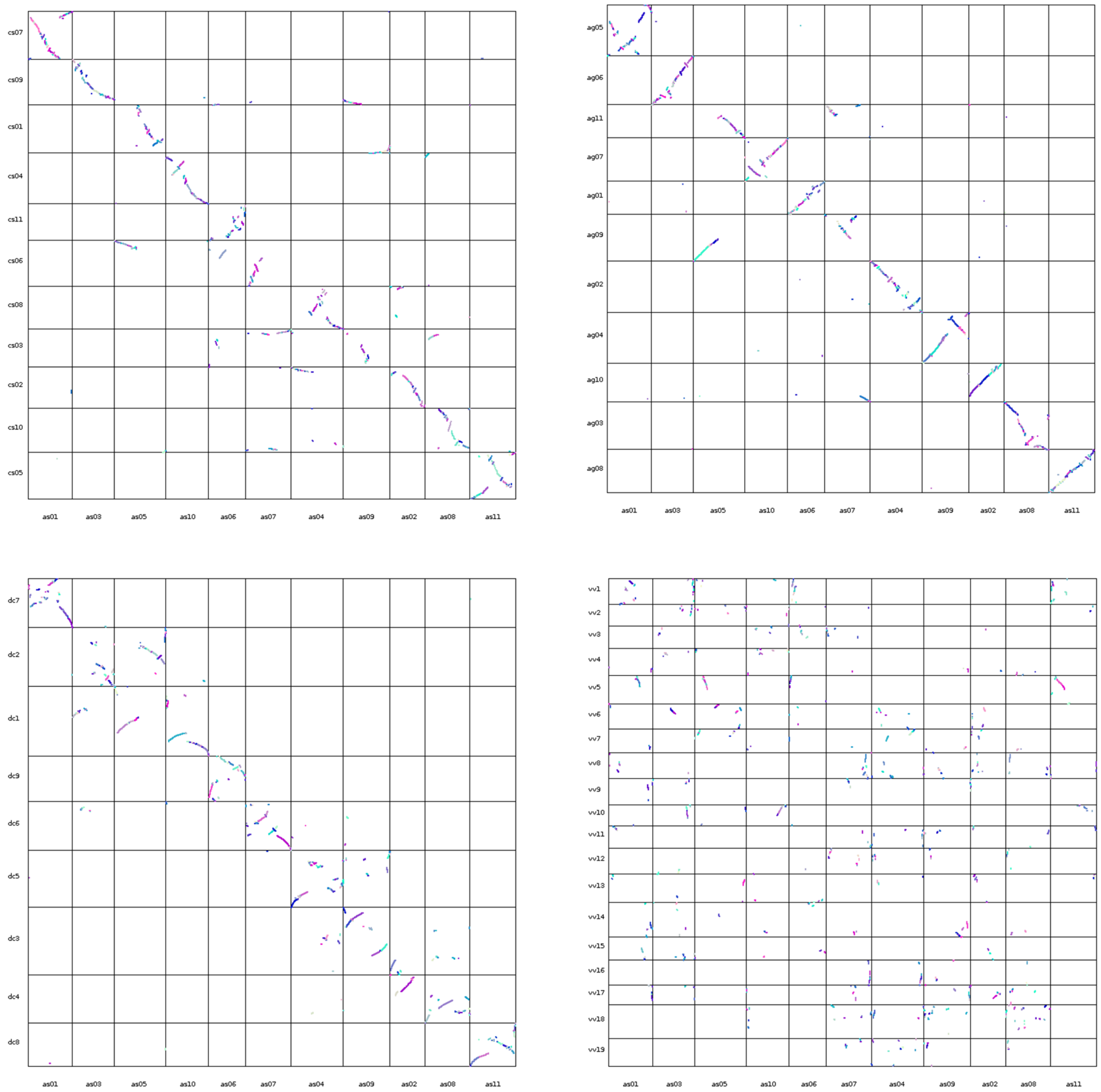

Figure S3. Homologous dot plot between *A. sinensis* and celery, carrot, coriander and grape genomes.

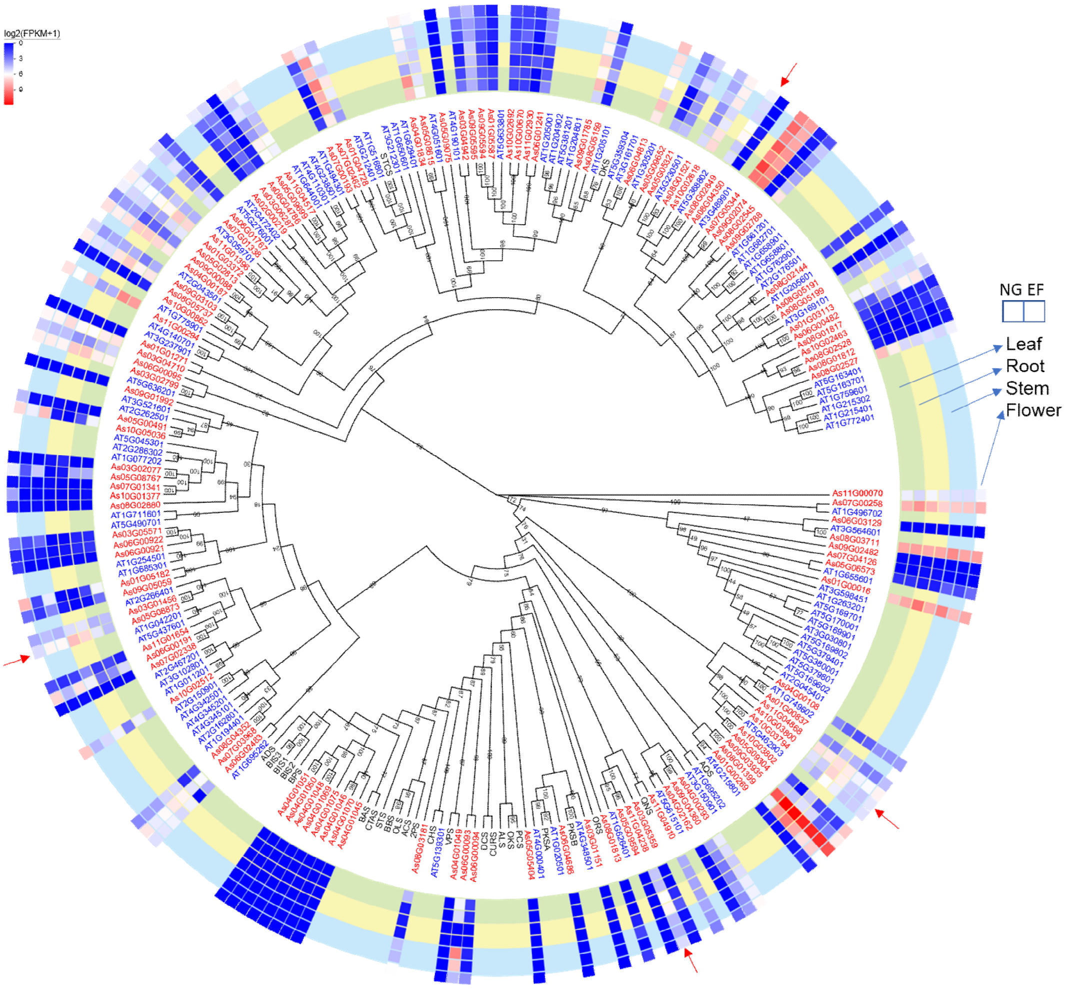

Figure S4. The phylogenetic tree of PKS genes and its expressions in different tissues (leaf, root, stem and flower) of *A. sinensis* between EF and NG plants. The red arrows indicated the specifically high expressed gene in *A. sinensis* root, including *As05G08873*, *As11G04238*, *As10G03800* and *As08G02849*.

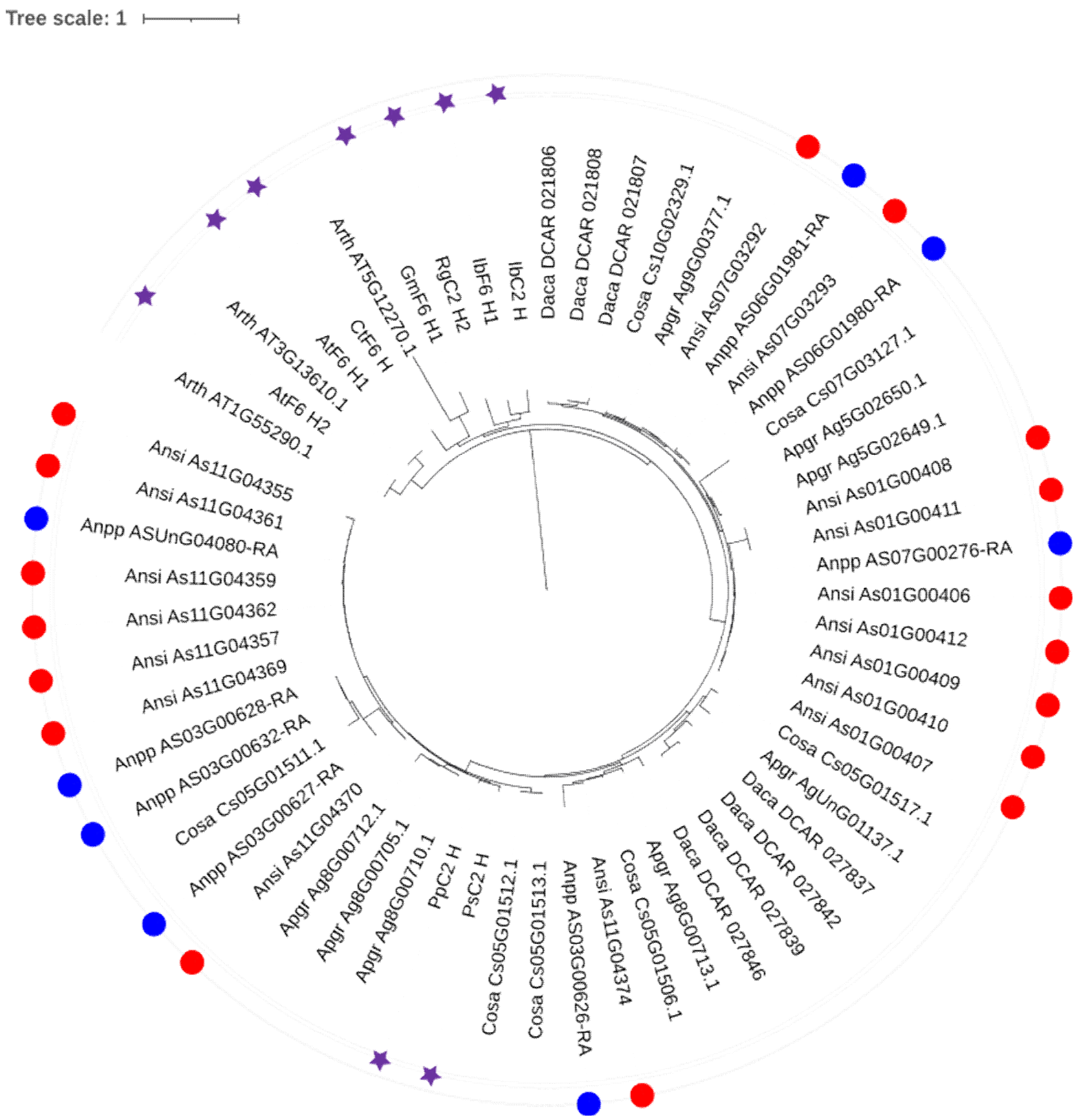

Figure S5. The phylogenetic tree of C2'H and F6'H in the typical Apiaceae species. The seed sequences were highlighted with purple stars and the red and blue dots indicated the genes from *A. sinensis* (QH) and *A. sinensis* (GS).

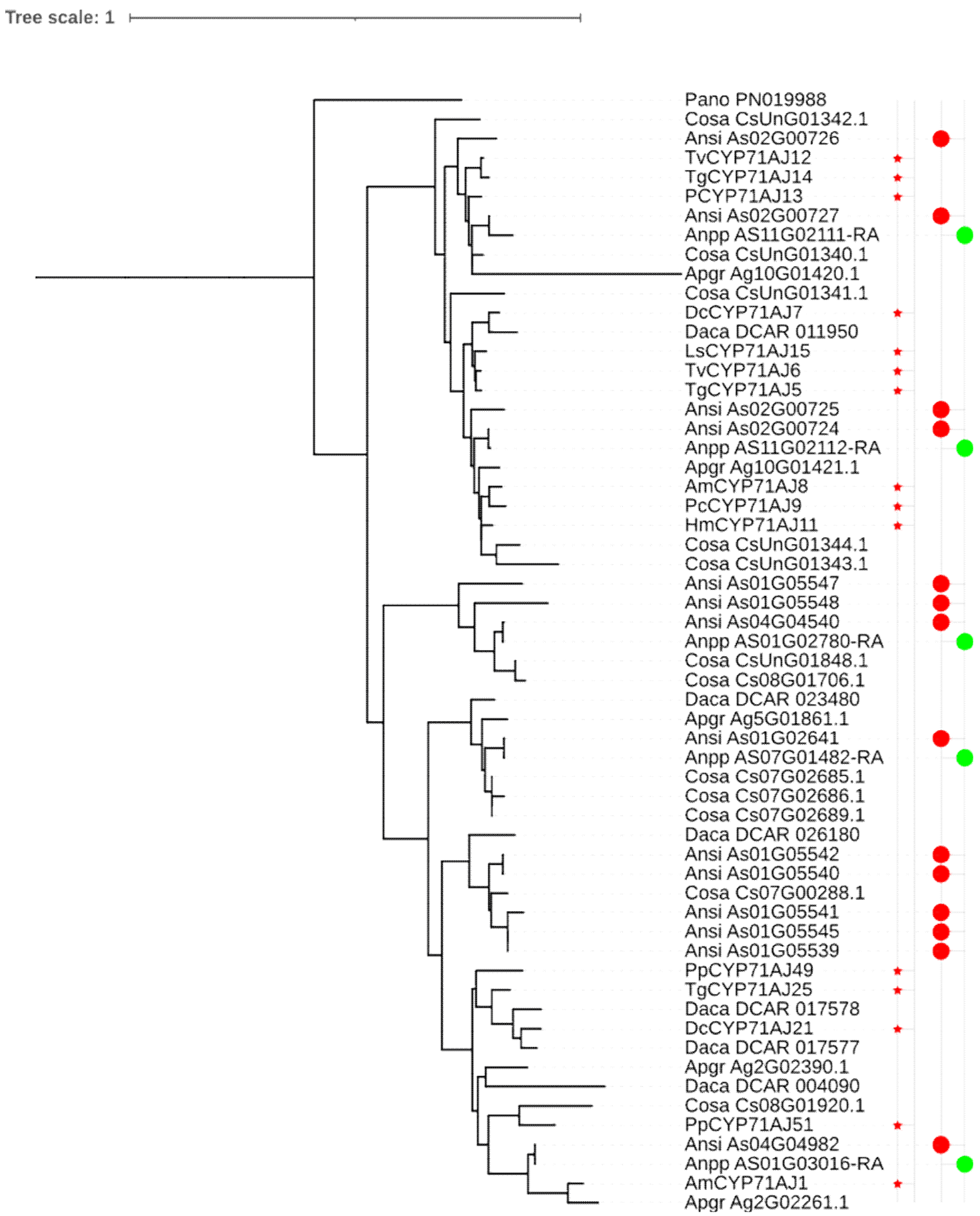

Figure S6. The phylogenetic tree of CYP71AJ genes in the typical Apiaceae species. The seed sequences were highlighted with red stars and the red and green dots indicated the genes from *A. sinensis* (QH) and *A. sinensis* (GS). It was obvious that there were much more CYP71AJ genes in *A. sinensis* (QH) than that in *A. sinensis* (GS)

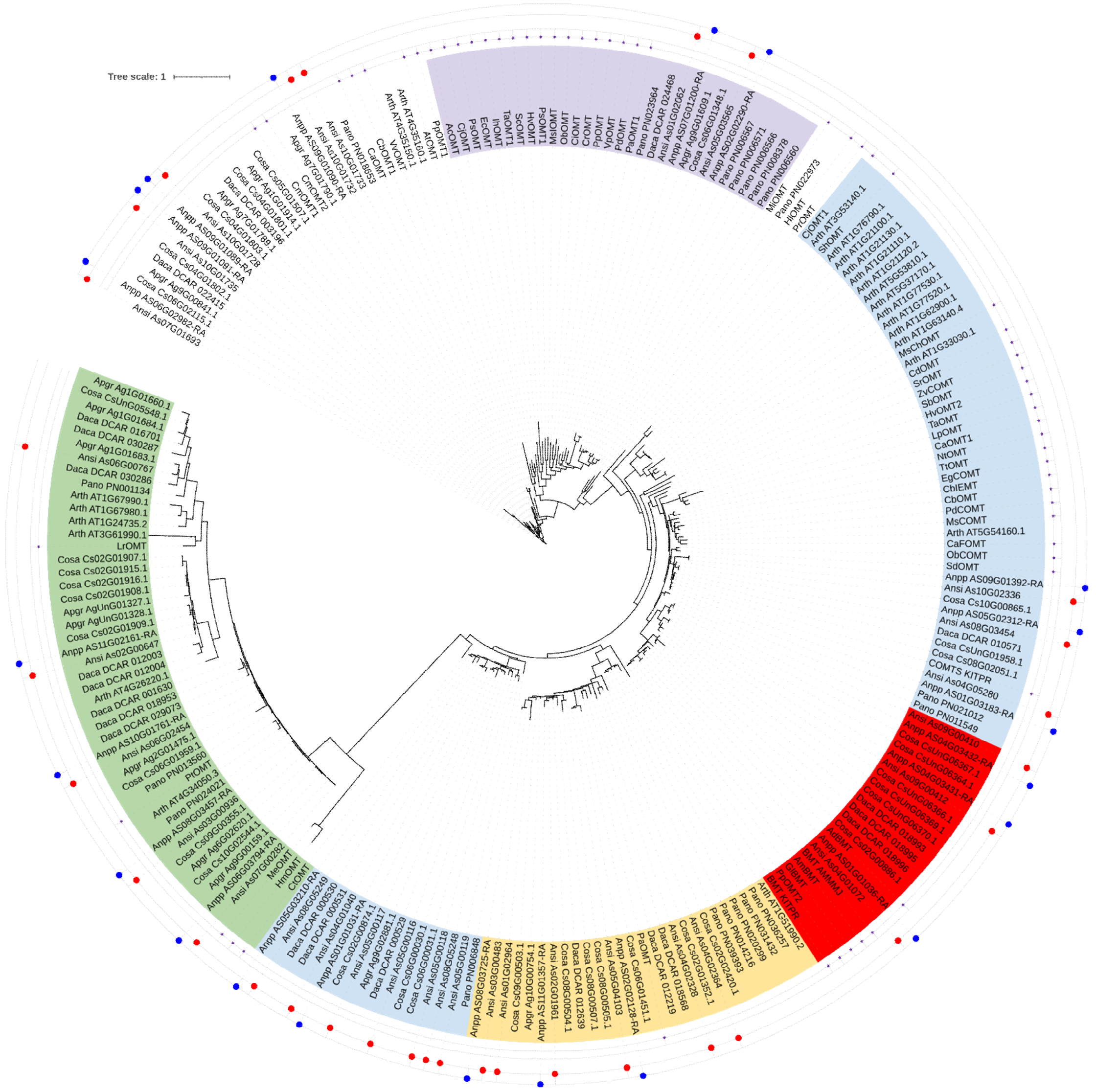

Figure S7. The phylogenetic tree of O-MT gene families in the typical Apiaceae species. The seed sequences were highlighted with purple stars and the red and blue dots indicated the genes from *A. sinensis* (QH) and *A. sinensis* (GS). BMT genes were highlighted with the red background.

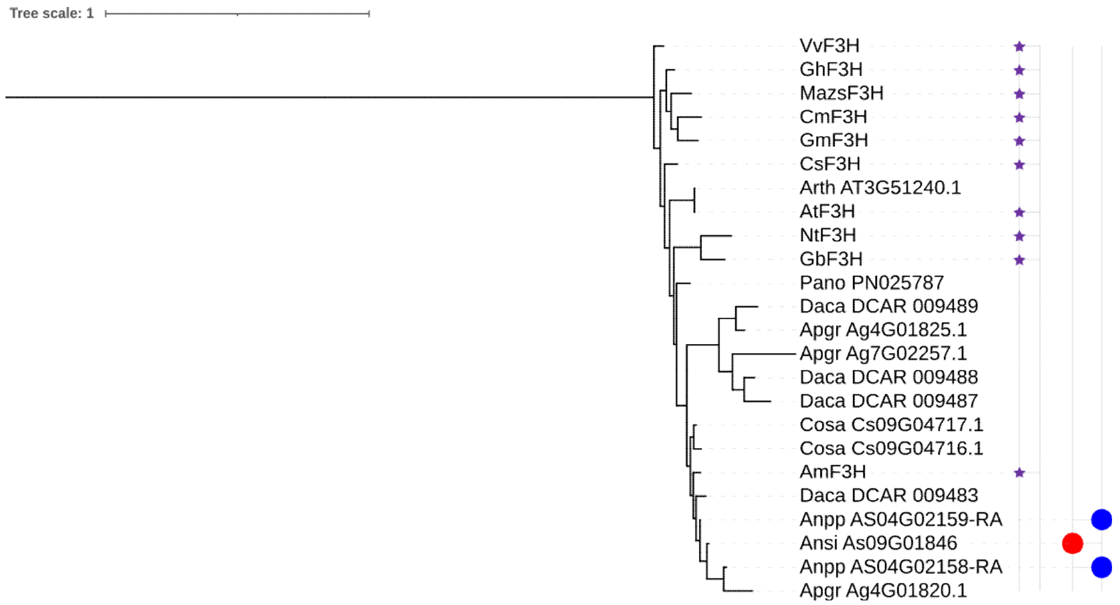

Figure S8. The phylogenetic tree of F3H in the typical Apiaceae species. The seed sequences were highlighted with purple stars and the red and blue dots indicated the genes from *A. sinensis* (QH) and *A. sinensis* (GS).

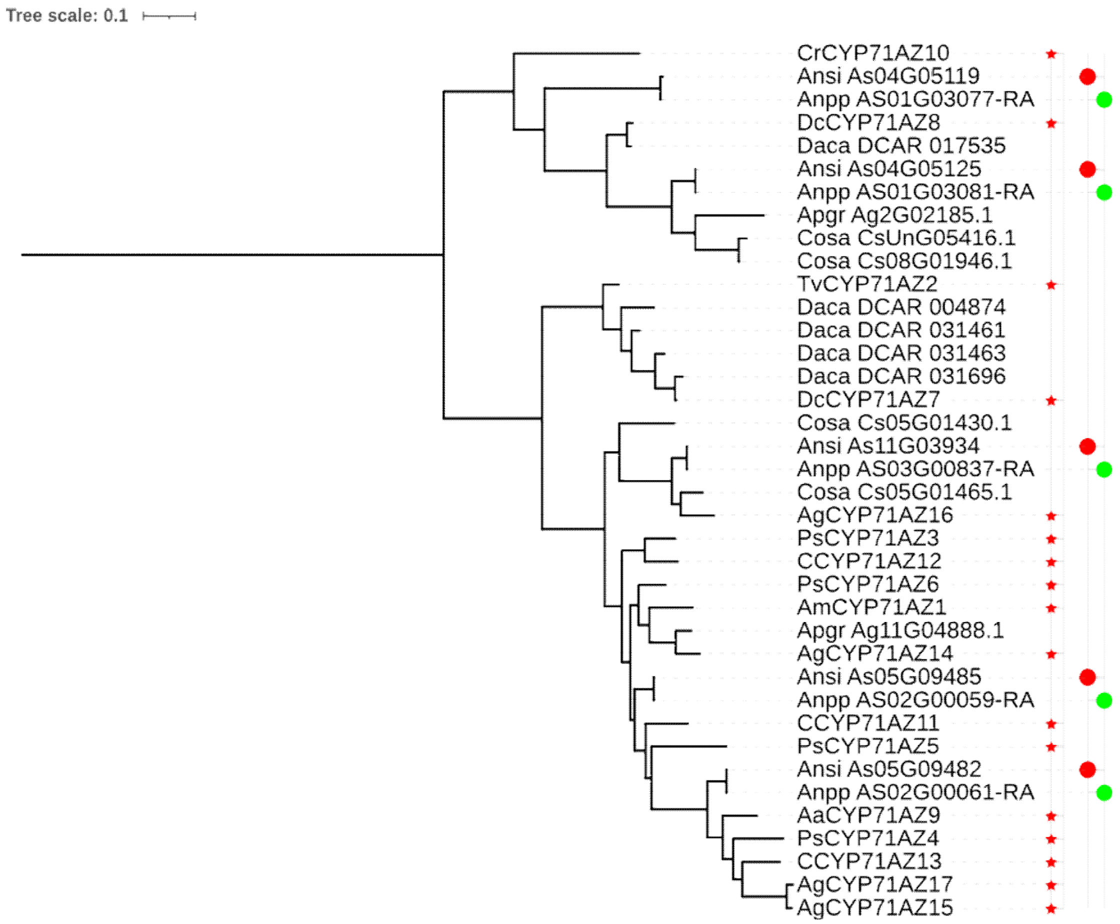

Figure S9. The phylogenetic tree of CPY71AZ gene families in the typical Apiaceae species. The seed sequences were highlighted with red stars and the red and green dots indicated the genes from *A. sinensis* (QH) and *A. sinensis* (GS).

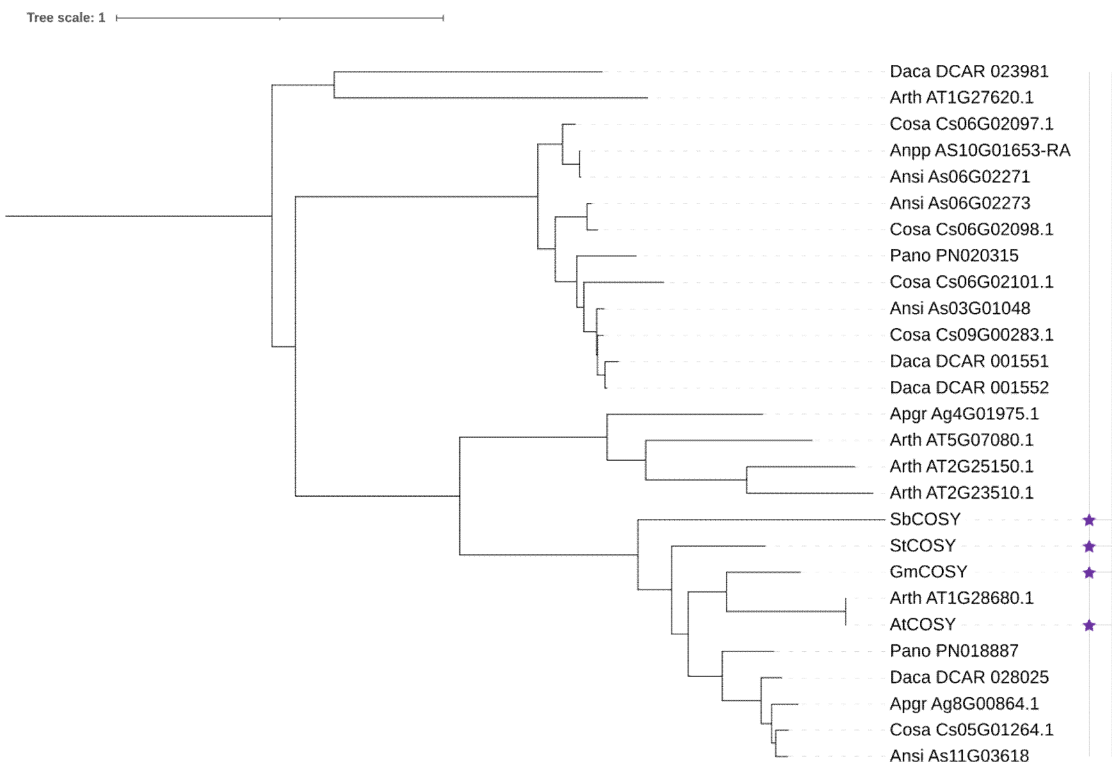

Figure S10. The phylogenetic tree of COSY in the typical Apiaceae species. The seed sequences were highlighted with purple stars and only one gene, *As11G03618* was identified as COSY gene from *A. sinensis* (QH).

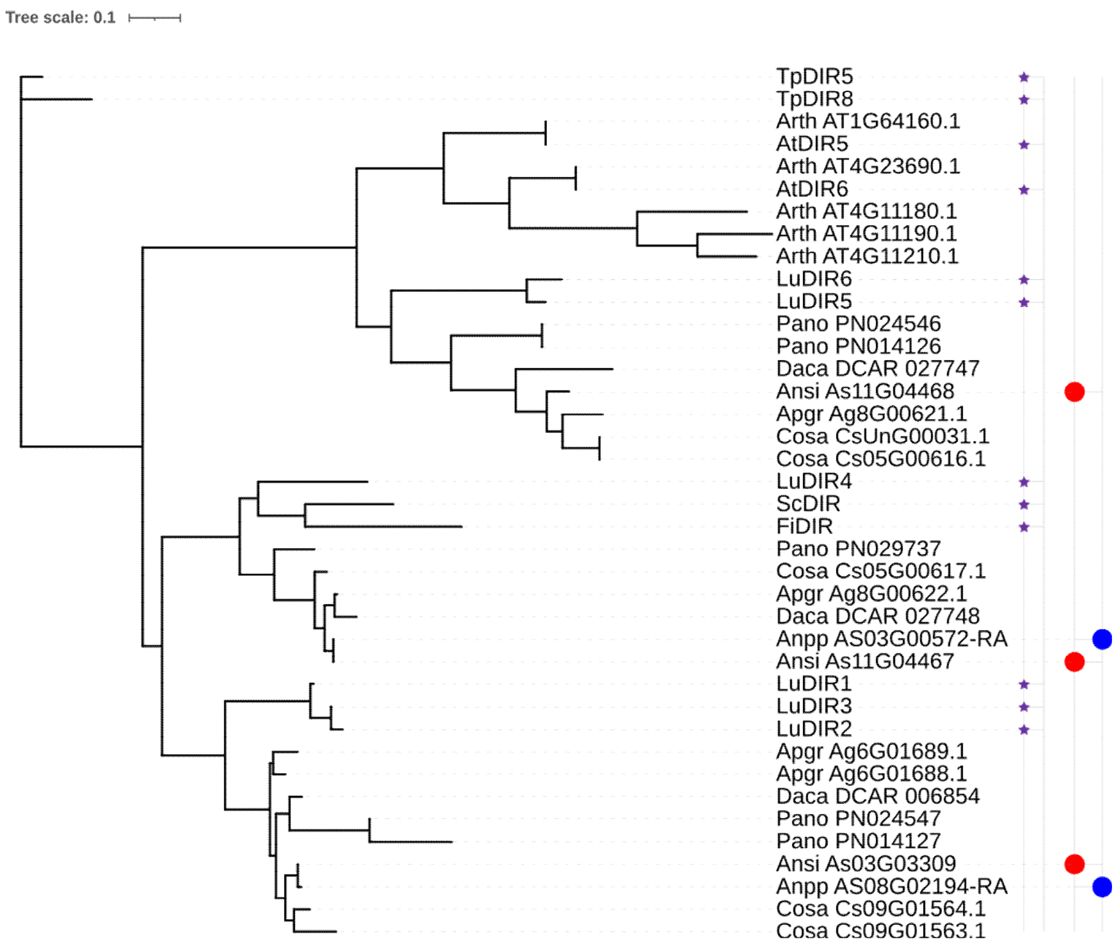

Figure S11. The phylogenetic tree of DIR genes in the typical Apiaceae species. The seed sequences were highlighted with purple stars and the red and blue dots indicated the genes from *A. sinensis* (QH) and *A. sinensis* (GS).

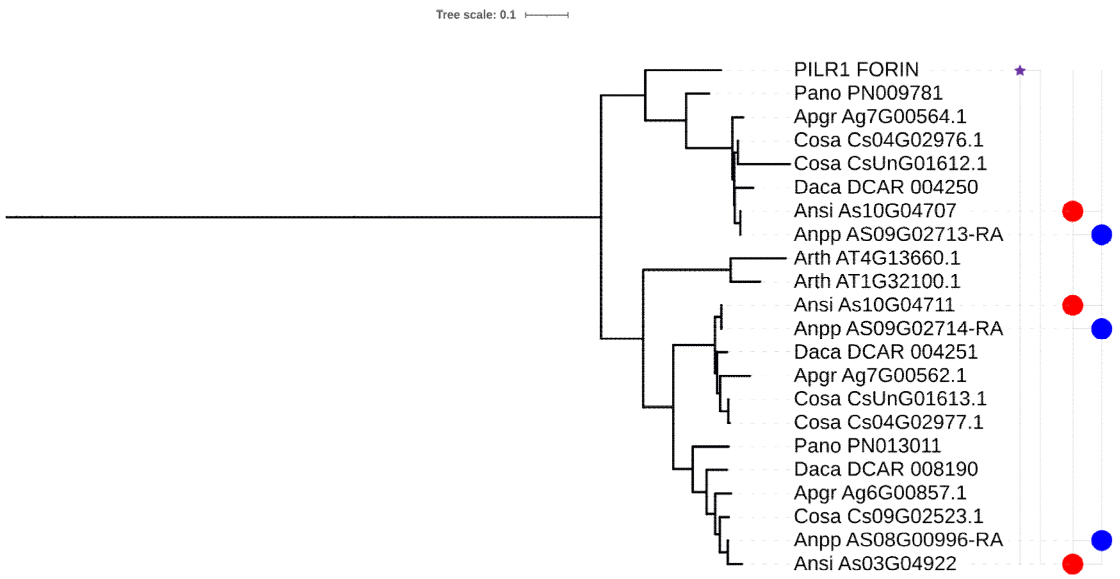

Figure S12. The phylogenetic tree of PLR genes in the typical Apiaceae species. The seed sequences were highlighted with purple stars and the red and blue dots indicated the genes from *A. sinensis* (QH) and *A. sinensis* (GS).

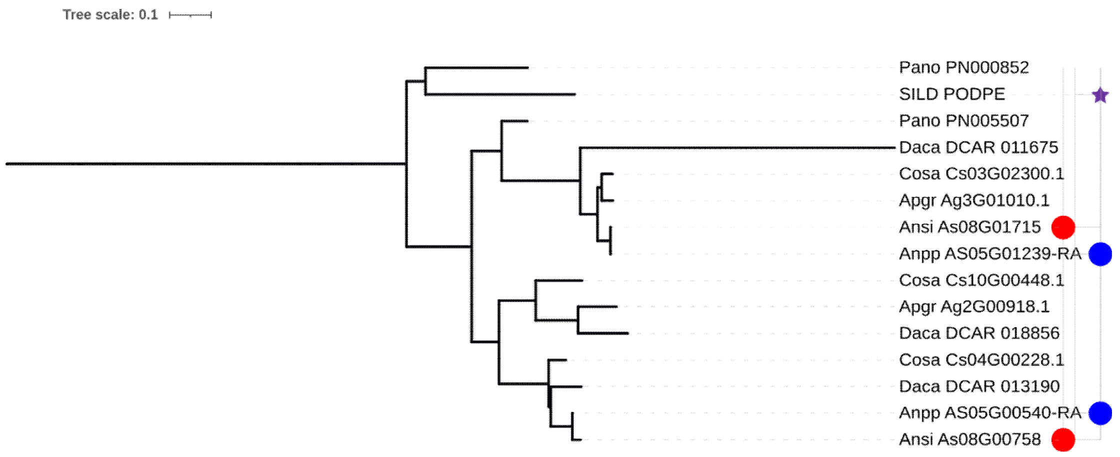

Figure S13. The phylogenetic tree of SIRD genes in the typical Apiaceae species. The seed sequences were highlighted with purple stars and the red and blue dots indicated the genes from *A. sinensis* (QH) and *A. sinensis* (GS).

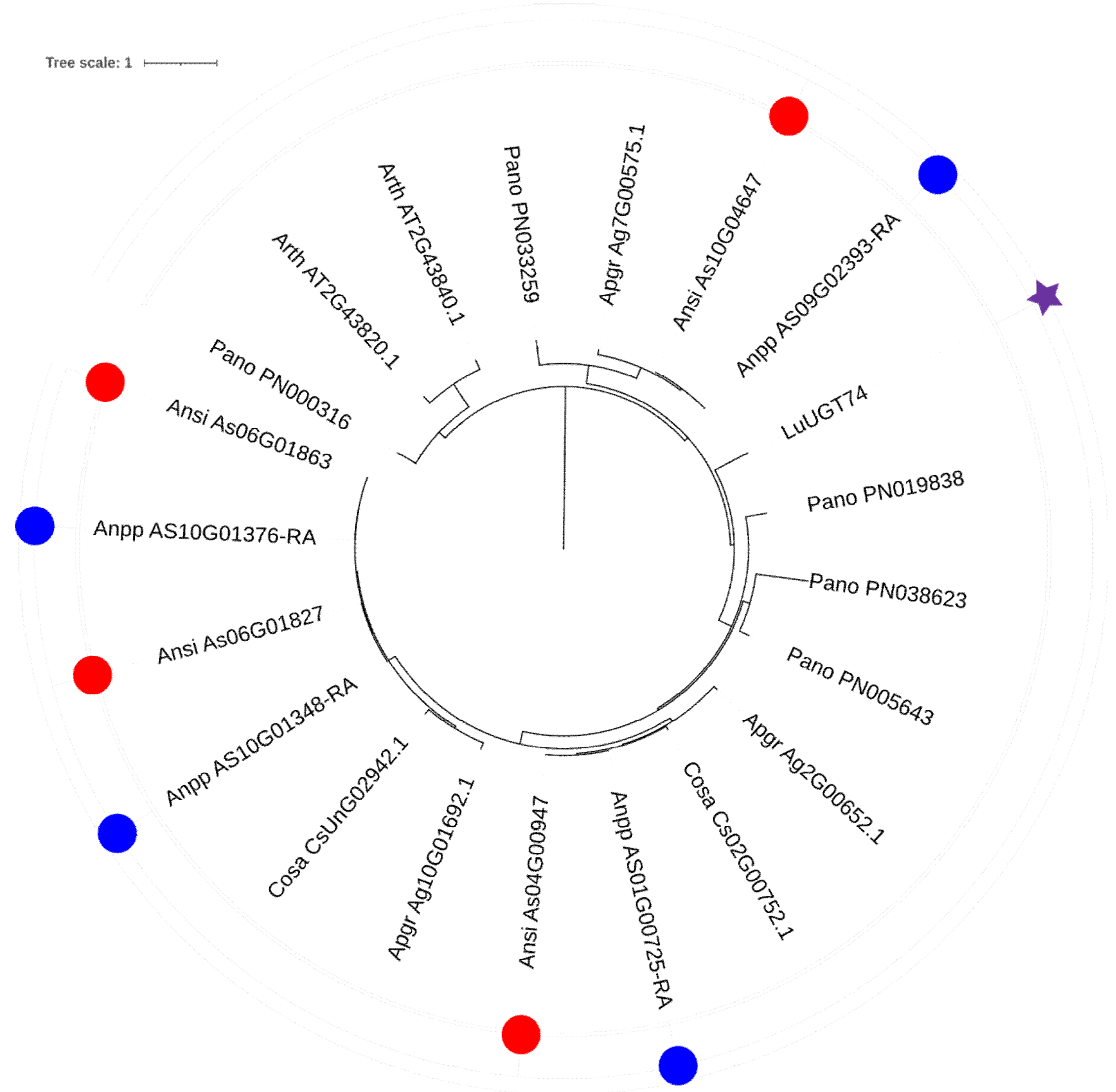

Figure S14. The phylogenetic tree of UGT74 genes in the typical Apiaceae species. The seed sequences were highlighted with purple stars and the red and blue dots indicated the genes from *A. sinensis* (QH) and *A. sinensis* (GS).

Table S1. List of sequencing data generated.

Type	Library	Platform	Insert size(bp)	Read length (bp)	Clean reads (M)	Clean data (Gp)	Clean coverage (x)
Genome	PCR-free	Illumina	350	150_150	-	60.77	21
	Single molecule	PacBio	-	8782*	-	376.43	132
	Hi-C	Illumina	-	150_150	-	324.98	114
RNAseq	flower_1	Illumina	250	150_150	47.88	7.18	-
	flower_2	Illumina	250	150_150	45.25	6.79	-
	flower_3	Illumina	250	150_150	49.56	7.43	-
	leaf_EF_1	Illumina	250	150_150	45.08	6.76	-
	leaf_EF_2	Illumina	250	150_150	48.1	7.22	-
	leaf_EF_3	Illumina	250	150_150	43.33	6.5	-
	leaf_NG_1	Illumina	250	150_150	52.06	7.81	-
	leaf_NG_2	Illumina	250	150_150	45.44	6.82	-
	leaf_NG_3	Illumina	250	150_150	45.86	6.88	-
	root_EF1	Illumina	250	150_150	44.54	6.68	-
	root_EF2	Illumina	250	150_150	43.69	6.55	-
	root_EF3	Illumina	250	150_150	42.32	6.35	-
	root_NG1	Illumina	250	150_150	43.4	6.51	-
	root_NG2	Illumina	250	150_150	48.35	7.25	-
	root_NG3	Illumina	250	150_150	45.44	6.82	-
	stem_EF_1	Illumina	250	150_150	43.67	6.55	-
	stem_EF_2	Illumina	250	150_150	43.13	6.47	-
	stem_EF_3	Illumina	250	150_150	43.22	6.48	-
	stem_NG1	Illumina	250	150_150	49.5	7.42	-
	stem_NG2	Illumina	250	150_150	44.07	6.61	-
	stem_NG3	Illumina	250	150_150	44.77	6.72	-
	stem	Illumina	250	150_150	49.29	7.63	-
	seed	Illumina	250	150_150	46.52	7.23	-
	root	Illumina	250	150_150	42.63	6.59	-
	leaf	Illumina	250	150_150	45.89	7.1	
	Total	-	-	-	1142.99	172.36	-

* The average length of PacBio reads.

Table S2. Genome length distribution results.

Chromosome name	Chromosome length	Chromosome length (%)	Gene number	Gene percentage (%)	Gene numbers per MB
Chr01	193,656,776	8.98	4101	9.99	21.18
Chr02	152,020,571	7.05	2901	7.07	19.08
Chr03	184,768,007	8.57	4203	10.24	22.75
Chr04	228,427,485	10.6	4222	10.29	18.48
Chr05	224,921,384	10.43	4815	11.73	21.41
Chr06	161,079,071	7.47	2978	7.26	18.49
Chr07	200,643,672	9.31	3927	9.57	19.57
Chr08	198,273,161	9.2	3631	8.85	18.31
Chr09	205,368,775	9.53	3621	8.82	17.63
Chr10	189,024,495	8.77	3149	7.67	16.66
Chr11	199,558,849	9.26	2811	6.85	14.09
ChrUn	18,011,198	0.84	681	1.66	37.81
Total	2,155,753,444	100	41040	100	19.04

Table S3. Genome assembly completeness evaluation with BUSCO groups.

	Genome		Gene set		HC		LC		A. sinensis (PJ) (Genome)		A. sinensis (PJ) (Gene set)	
	Number	%	Number	%	Number	%	Number	%	Number	%	Number	%
Complete	1600	99.13	1556	96.41	1545	95.72	13	0.81	1581	98.0	1421	88.1
Complete single-copy	1483	91.88	1451	89.9	1441	89.28	13	0.81	1465	90.8	1333	82.6
Complete duplicated	117	7.25	105	6.51	104	6.44	0	0	116	7.2	88	5.5
Fragmented	6	0.37	26	1.61	25	1.55	1	0.06	6	0.4	51	3.2
Missing	8	0.5	32	1.98	44	2.73	1600	99.13	27	1.6	142	8.7

BUSCO version is: 5.1.0

The lineage dataset is: (Creation date: 2020-09-10, number of genomes: 50, number of BUSCOs: 1614)

Table S4. Repeat element in the *A. sinensis* genome.

Type	Repbase TEs		TE protiens		De novo		Cobpined TEs	
	Length (bp)	% in genome	Length (bp)	% in genome	Length (bp)	% in genome	Length (bp)	% in genome
DNA	10,347,805	0.48	1,782,222	0.08	78,593,047	3.65	82,512,686	3.83
LINE	4,981,767	0.23	3,315,293	0.15	27,054,485	1.26	28,709,115	1.33
SINE	104,316	0	-	0	668,157	0.03	764,618	0.04
LTR	343,481,024	15.94	454,548,447	21.09	1,259,715,753	58.45	1,276,667,829	59.24
Other	64	0	-	0	-	0	64	0
Unknown	-	0	-	0	426,107,135	19.77	426,107,135	19.77
Total	358,539,068	16.64	459,644,637	21.33	1,710,377,864	79.36	1,729,298,676	80.24

Table S5. Repeat elements identified by De novo method in the *A. sinensis* genome.

Type		Length(bp)	Percentage(%)
LTR	Total	1,259,715,753	58.45
	LTR/Copia	889,860,568	41.29
	LTR/Gypsy	348,214,567	16.16
	LTR/Caulimovirus	7,292,125	0.34
	LTR/Caulimoviru	2,782,320	0.13
	LTR/Pao	1,299,061	0.06
	LTR/ERV1	833,471	0.04
	LTR/ERVK	582,108	0.03
	LTR/LTR	8,624,974	0.4
	LTR/Cassandra	65,381	0
	Other	161,178	0.01
DNA transposons		78,593,047	3.65
Simple_repeat		93,187	0
Satellite		813,539	0.04
LINE		27,054,485	1.26
SINE		668,157	0.03
Unknown		426,107,135	19.77
Total		1,710,377,864	79.36

Table S6. Basic statistical results of gene structure prediction.

Gene set type		Gene number	Average gene length (bp)	Average CDS length (bp)	Average exon number	Average exon length (bp)	Average intron length (bp)
Homolog	AG	44,606	3121.42	968.6	3.98	243.25	721.96
	AT	35,260	2782.41	988.43	3.92	252.18	614.46
	CS	61,617	2884.81	862.4	3.56	242.23	789.91
	DC	50,461	3149.39	918.14	3.72	247.08	821.53
	GM	46,950	2508.23	885.96	3.46	255.78	658.46
	HA	47,156	3076.11	1024.15	3.48	293.99	826.22
	LS	36,804	3233.61	979.68	4.01	244.43	749.29
	OS	37,383	2713.57	942.95	3.57	264.05	688.67
	SL	42,671	2936.92	911.02	3.65	249.7	764.94
	VV	37,660	3104.8	979.54	3.96	247.1	717
Denovo	augustus	57,229	3765.11	1003.07	4.2	239.01	864
	glimmerHMM	126,850	15505.7	598.75	3.26	183.82	6603.94
	SNAP	96,693	5863.36	648.75	4.08	158.85	1690.85
RNAseq	Cufflinks	37,158	5418.04	1224.03	4.84	252.65	628.2
Final	EVM	68,807	3293.49	877.07	3.75	234.12	879.88
	HC	41,040	3951.49	1126.57	4.69	240.09	765.08
	LC	27,767	2320.96	508.32	2.35	216.48	1344.62

Abbreviation: AG: Apium graveolens; AT: Arabidopsis thaliana; CS: Coriandrum sativum; DC: Daucus carota; GM: Glycine max; HA: Helianthus annuus; LS: Lactuca sativa; OS: Oryza sativa; SL: Solanum lycopersicum; VV: Vitis vinifera.

Table S7. The 20 species used to construct phylogenetic tree.

**Species**	**order**	**family**	**Assembly level**	**shade plant**	**UTR**
Vitis vinifera	Vitales	Vitaceae	chromosome	no	PhytozomeV13
Populus trichocarpa	Malpighiales	Salicaceae	chromosome	no	PhytozomeV13
Cannabis sativa	Rosales	Cannabaceae	chromosome	no	GCF_900626175.2(NCBI)
Arabidopsis thaliana	Brassicales	Brassicaceae	chromosome	no	TAIR10/Araport11(Phytozome)
Theobroma cacao	Malvales	Malvaceae	chromosome	no	GCF_000208745.1(NCBI)
Camellia sinensis	Ericales	Theaceae	chromosome	no	GCF_004153795.1(NCBI)
Ophiorrhiza pumila	Gentianales	Rubiaceae	chromosome	yes	http://pumila.kazusa.or.jp/
Antirrhinum majus	Lamiales	Plantaginaceae	chromosome	no	http://bioinfo.sibs.ac.cn/Am/download_v2.php
Salvia miltiorrhiza	Lamiales	Lamiaceae	chromosome	no	https://ngdc.cncb.ac.cn/search/?dbId=gwh&q=PRJCA003150
Scutellaria baicalensis	Lamiales	Lamiaceae	chromosome	no	https://ngdc.cncb.ac.cn/gwh/Genome/813/show
Solanum lycopersicum	Solanales	Solanaceae	chromosome	no	GCF_000188115.4(NCBI)
Artemisia annua	Asterales	Asteraceae	scaffold	no	GCA_003112345.1(NCBI)
Taraxacum mongolicum	Asterales	Asteraceae	chromosome	no	https://ngdc.cncb.ac.cn/gwh/Assembly/19733/show
Panax ginseng	Apiales	Araliaceae	scaffold	yes	http://ginsengdb.snu.ac.kr/data.php

Panax notoginseng	Apiales	Araliaceae	chromosome	yes	https://ftp.cngb.org/pub/CNSA/data2/CNP0001042/CNS0223752/CNA0013972/
Daucus carota	Apiales	Apiaceae	chromosome	no	PhytozomeV13
Apium graveolens	Apiales	Apiaceae	chromosome	no	(CGD: http://celerydb.bio2db.com)
Coriandrum sativum	Apiales	Apiaceae	chromosome	no	CGDB (http://cgdb.bio2db.com).
Angelica sinensis	Apiales	Apiaceae	chromosome	yes	This study
Lonicera japonica	Dipsacales	Caprifoliaceae	chromosome	no	https://ngdc.cncb.ac.cn/search/?dbId=gwh&q=PRJCA001719

Table S8. Gene family clustering of five Apiaceae species and one outer species (*P. notoginseng*) by Orthofinder.

Types	Numbers	Percent(%)
Species used	6	-
Total genes	226299	-
Genes in orthogroups	206682	91.3
Unassigned genes	19617	8.7
Orthogroups	29108	-
Species-specific orthogroups	4087	-
Genes in species-specific orthogroups	16378	7.2
Orthogroups with all species present	11519	-
Single-copy orthogroups	3189	-

Table S9. KEGG gene family expansion analysis in *A. sinensis* genome.

#Pathway	Asi-expand(P0.05) (2910)	All-gene (28974)	Pvalue	Qvalue	Pathway ID	Level 1	Level 2	Enrich_factor
Oxidative phosphorylation	151	472	6.57E-40	8.22E-38	ko00190	Metabolism	Energy metabolism	0.3199
Photosynthesis - antenna proteins	25	44	2.12E-14	1.11E-12	ko00196	Metabolism	Energy metabolism	0.5682
Zeatin biosynthesis	50	155	2.79E-14	1.11E-12	ko00908	Metabolism	Metabolism of terpenoids and polyketides	0.3226
Photosynthesis	73	285	3.57E-14	1.11E-12	ko00195	Metabolism	Energy metabolism	0.2561
Metabolic pathways	813	6555	9.17E-13	2.29E-11	ko01100	Metabolism	Global and overview maps	0.1240
Protein processing in endoplasmic reticulum	149	829	1.21E-12	2.52E-11	ko04141	Genetic Information Processing	Folding, sorting and degradation	0.1797
Biosynthesis of secondary metabolites	450	3363	2.50E-11	3.94E-10	ko01110	Metabolism	Global and overview maps	0.1338
Sesquiterpenoid and triterpenoid biosynthesis	39	122	2.52E-11	3.94E-10	ko00909	Metabolism	Metabolism of terpenoids and polyketides	0.3197
Phenylpropanoid biosynthesis	122	685	2.46E-10	3.41E-09	ko00940	Metabolism	Biosynthesis of other secondary metabolites	0.1781
Fatty acid metabolism	61	264	4.18E-10	5.22E-09	ko01212	Metabolism	Global and overview maps	0.2311

Biosynthesis of unsaturated fatty acids	34	108	7.36E-10	8.36E-09	ko01040	Metabolism	Lipid metabolism	0.3148
Cyanoamino acid metabolism	67	345	1.02E-07	1.06E-06	ko00460	Metabolism	Metabolism of other amino acids	0.1942
Selenocompound metabolism	26	91	6.03E-07	5.80E-06	ko00450	Metabolism	Metabolism of other amino acids	0.2857
Tryptophan metabolism	43	198	9.12E-07	8.14E-06	ko00380	Metabolism	Amino acid metabolism	0.2172
Endocytosis	110	711	3.05E-06	2.54E-05	ko04144	Cellular Processes	Transport and catabolism	0.1547
Spliceosome	98	653	3.56E-05	2.78E-04	ko03040	Genetic Information Processing	Transcription	0.1501
Carbon fixation in photosynthetic organisms	53	325	0.000286	2.10E-03	ko00710	Metabolism	Energy metabolism	0.1631
Carbon metabolism	104	755	0.000568	3.94E-03	ko01200	Metabolism	Global and overview maps	0.1377
Benzoxazinoid biosynthesis	9	28	0.001201	7.90E-03	ko00402	Metabolism	Biosynthesis of other secondary metabolites	0.3214
Pyruvate metabolism	36	219	0.002156	1.29E-02	ko00620	Metabolism	Carbohydrate metabolism	0.1644
Flavonoid biosynthesis	33	196	0.002169	0.0129	ko00941	Metabolism	Biosynthesis of other secondary metabolites	0.1684
Starch and sucrose metabolism	97	727	0.00233	0.0132	ko00500	Metabolism	Carbohydrate metabolism	0.1334
Betalain biosynthesis	13	56	0.003117	0.0169	ko00965	Metabolism	Biosynthesis of other secondary metabolites	0.2321

Propanoate metabolism	23	134	0.007425	0.0374	ko00640	Metabolism	Carbohydrate metabolism	0.1716
Flavone and flavonol biosynthesis	12	55	0.007479	0.0374	ko00944	Metabolism	Biosynthesis of other secondary metabolites	0.2182
Glucosinolate biosynthesis	12	56	0.008669	0.0417	ko00966	Metabolism	Biosynthesis of other secondary metabolites	0.2143
Isoflavonoid biosynthesis	11	50	0.009591	0.0437	ko00943	Metabolism	Biosynthesis of other secondary metabolites	0.2200
Indole alkaloid biosynthesis	8	31	0.009794	0.0437	ko00901	Metabolism	Biosynthesis of other secondary metabolites	0.2581
Fatty acid biosynthesis	22	137	0.018268	0.0783	ko00061	Metabolism	Lipid metabolism	0.1606
Glycosphingolipid biosynthesis - lacto and neolacto series	4	11	0.018789	0.0783	ko00601	Metabolism	Glycan biosynthesis and metabolism	0.3636
Tyrosine metabolism	21	132	0.022823	0.0920	ko00350	Metabolism	Amino acid metabolism	0.1591
alpha-Linolenic acid metabolism	21	136	0.030667	0.1200	ko00592	Metabolism	Lipid metabolism	0.1544
Ubiquinone and other terpenoid-quinone biosynthesis	22	146	0.034994	0.1330	ko00130	Metabolism	Metabolism of cofactors and vitamins	0.1507

Table S10. The detailed peak values of KS distributions in Figure 2e.

	Peak1	Peak2	Peak3
Ansi vs Ansi	0.018	0.47	0.94
Anpp vs Anpp	0.014	0.48	0.95
Cosa vs Cosa	0.027	0.508	0.966
Agpr vs Agpr	0.03	0.557	1.01
Daca vs Daca	NA	0.64	1.09
Vivi vs Vivi	NA	NA	1.2

Abbreviation: Ansi: *A. sinensis* (QH); Anpp: A. sinensis (GS); Cosa: *Coriandrum sativum*; Apgr: *Apium graveolens*; Daca: *Daucus carota*; Vivi: *Vitis vinifera*.

Table S11 The SNP and INDEL distribution among the chromosomes between *A. sinensis* (PJ) and *A. sinensis* (this study) genome.

#chr	Len	SNP_numbers	SNP_desity(%)	Indel_numbers	Indel_desity(%)
Chr01	193656776	83838	0.0433	17823	0.0092
Chr02	152020571	69841	0.0459	14849	0.0098
Chr03	184768007	93994	0.0509	21130	0.0114
Chr04	228427485	135570	0.0593	27104	0.0119
Chr05	224921384	151310	0.0673	26602	0.0118
Chr06	161079071	75517	0.0469	15877	0.0099
Chr07	200643672	110224	0.0549	21474	0.0107
Chr08	198273161	102715	0.0518	21035	0.0106
Chr09	205368775	98661	0.048	19986	0.0097
Chr10	189024495	134700	0.0713	25401	0.0134
Chr11	199558849	147116	0.0737	26736	0.0134

Table S12. The gene numbers of *ACC* genes in *A. sinensis (QH), A. sinensis (GS)* and other 8 related species annotated using KEGG database.

Gene name	KO term	total	Ansi	Anpp	Cosa	Apgr	Daca	Pano	Soly	Potr	Arth	Vivi	annoation
CAC2	K01961	23	2	2	4	3	2	5	1	2	1	1	acetyl-CoA carboxylase, biotin carboxylase subunit [EC:6.4.1.2 6.3.4.14]
CAC3	K01962	28	3	3	5	3	2	4	2	3	1	2	acetyl-CoA carboxylase carboxyl transferase subunit alpha [EC:6.4.1.2 2.1.3.15]
accD	K01963	55	10	31	2	0	2	3	5	1	1	0	acetyl-CoA carboxylase carboxyl transferase subunit beta [EC:6.4.1.2 2.1.3.15]
BCCP2,CAC1	K02160	30	4	4	3	3	3	3	2	3	2	3	acetyl-CoA carboxylase biotin carboxyl carrier protein
ACC1,ACC2	K11262	18	1	1	4	1	1	3	1	3	2	1	acetyl-CoA carboxylase / biotin carboxylase 1 [EC:6.4.1.2 6.3.4.14 2.1.3.15]
		154	20	41	18	10	10	18	11	12	7	7	

Abbreviation: Ansi: *A. sinensis* (QH); Anpp: A. sinensis (GS); Cosa: *Coriandrum sativum*; Apgr: *Apium graveolens*; Daca: *Daucus carota*; Pano: *Panax notoginseng*; Soly: *Solanum lycopersicum*; Potr: *Populus trichocarpa*; Arth: *Arabidopsis thaliana*; Vivi: *Vitis vinifera*.

Table S13. The gene numbers of *PKS* genes in *A. sinensis* (QH), *A. sinensis* (GS) annotated using the PKS genes reviewed by Bisht et al. 2021 Frontiers in Plant Science.

type III PKSs from each cyclization class	ID	GeneSymbol	GeneName	Ref_Reviewed by Bisht et al. 2021 Frontiers in Plant Science	*A. sinensis* (QH) (this study)	*A. sinensis* (GS)
Alodl cyclization	5	STCS	Stilbenecarboxylate synthase (STCS)	24	55	53
Alodl cyclization	6	STS	**Stilbene synthase (STS)**	19	1	0
Alodl cyclization	7	ORS	2′ -oxoalkylresorcinol Synthase	4	7	8
Alodl cyclization	8	BIS	Biphenyl synthase (BIS)	11	0	0
Alodl cyclization	9	OLS	Olivetol synthase	21	0	0
Alodl cyclization	10	BBS	**bibenzyl synthase**	26	0	0
Alodl cyclization	11	ALS	Aloesone synthase (ALS)	22	-	0
Alodl cyclization	12	OKS	Octaketides synthase (OKS)	13	0	0
Claisen cyclization	14	PCS	pentaketide chromone synthase	5	0	0
Claisen cyclization	15	ACS	acridone synthase	17	2	4
Claisen cyclization	16	CHS	chalcone synthase	15	7	5
Claisen cyclization	17	QNS	quinolone synthase	18	4	2
Claisen cyclization	18	BPS	benzaphenone synthase	10	0	0
Claisen_cyclization	13	VPS	valerophenone synthase	20	7	5
lactonlization	1	CTAS	p-Coumaroyl triacetic acid synthase (CTAS)	25	0	0
lactonlization	2	2PS	2-Pyrone synthase (2PS)	27	7	6
lactonlization	3	PKSA	Type III polyketide synthase A (PKSA)	1	1	1

lactonlization	4	PKSB	Type III polyketide synthase B (PKSB)	3	4	4
Non-cyclization	19	QNS	quinolone synthase	18	-	
Non-cyclization	20	DKS	diketide synthase		24	22
Non-cyclization	21	BAS	benzalacetone synthase	23	1	0
Non-cyclization	22	DCS	diketide-CoA synthase	7	0	0
Non-cyclization	23	CURS	Curcumin synthase	8	0	0
Non-cyclization	24	ADS	alkyldiketide-CoA synthase	14	0	0
Non-cyclization	25	AQS	alkylquinolone synthase	12	0	0
Non-cyclization	26	AbYPKS	type III PKS from Atropa belladonna		-	-
Non-cyclization	27	HsPKS4	type III PKS from Huperzia serrata		-	-
lactonlization	28		Anther specific CHS like synthase	2		
Alodl cyclization	29		Orcinol Synthase	9		
Claisen cyclization	30		CHS-like polylketide synthase	6		
Claisen cyclization	31		Homoeriodictyol/eriodictyol CHS	16		
Non-cyclization	32		Pyrrolidine ketide synthase	28		
Total				424	120	110

Table S14. Seeds sequences information used for identification of PT genes.

#gene_names	Abbivations	Accession_ID	Organism	Function (Substrates)	Types
OsPPT1	PT	BAE96574.1	*Oryza sativa*		a
ZmPPT	PT	NP_001148558.1	*Zea mays*		a
AtPPT1	PT	NP_567688	*Arabidopsis thaliana*	*p*-hydroxybenzoic acid	a
LePGT-1	PT	BAB84122.1	*Lithospermum erythrorhizon*		a
LePGT-2	PT	BAB84123.1	*Lithospermum erythrorhizon*		a
GmVTE2-1	PT	ABB70126.1	*Glycine max rmum*		b
TaVTE2-1	PT	ABB70123.1	*Triticum aestivum*		b
ZmVTE2-1	PT	ABB70122.1	*Zea mays*		b
CpVTE2-1	PT	ABB70125.1	*Cuphea avigera*		b
AtVTE2-1	PT	AAM10489.1	*Arabidopsis thaliana*		b
ApVTE2-1	PT	ABB70124.1	*Allium ampeloprasum*		b
HvHGGT	PT	AAP43911.1	*Hordeum vulgare*		b
OsHGGT	PT	AAP43913.1	*Oryza sativa*	Homogentisate acid	b
TaHGGT	PT	AAP43912.1	*Triticum aestivum*		b
ZmHGGT	PT	XP_008659772.1	*Zea mays*		b
GmVTE2-2	PT	KRH71769.1	*Glycine max*		b
OsVTE2-2	PT	XP_015646905.1	*Oryza sativa*		b
ZmVTE2-2	PT	NP_001146703.1	*Zea mays*		b
DcVTE2-2	PT	XP_017246707.1	*Daucus carota*		b
AtVTE2-2	PT	ABB70127.1	*Arabidopsis thaliana*		b
AtrVTE2-2	PT	XP_011628799.1	*Amborella trichopoda*		b
GmATG4	PT	NP_001239633.1	*Glycine max*		c
OsATG4	PT	ABO31092.1	*Oryza sativa*	Chlorophyllide a/b	c
ZmATG4	PT	NP_001142204.1	*Zea mays*		c
AtATG4	PT	NP_190750.1	*Arabidopsis thaliana*		c
GmCOX10	PT	XP_003556552.1	*Glycine max*		d
OsCOX10	PT	EEC70799.1	*Oryza sativa*	Haem B	d
AtCOX10	PT	NP_566019.1	*Arabidopsis thaliana*		d
AtABC4	PT	NP_001117518.1	*Arabidopsis thaliana*		e
GmABC4	PT	XP_003532605.1	*Glycine max*	1,4-dihydroxy-2-napthoic acid	e
OsABC4	PT	NP_001049226.1	*Oryza sativa*		e
ZmABC4	PT	NP_001152170.1	*Zea mays*		e
PcPT	PT	BAO31627.1	*Petroselinum crispum*		f
PsPT1	PT	AJW31563.1	*Pastinaca sativa*	Coumarin	f
PsPT2	PT	AJW31564.1	*Pastinaca sativa*		f
ClPT1	PT	BAP27988.1	*Citrus limon*		f

FcPT1a	PT	BBC82715.1	*Ficus carica*		f
GmC4DT	PT	BAW32575.1	*Glycine max*		g
GmG2DT	PT	BAW32578.1	*Glycine max*		g
GmG4DT	PT	BAH22520.1	*Glycine max*		g
GmIDT1	PT	BAW32576.1	*Glycine max*		g
GmIDT2	PT	BAW32577.1	*Glycine max*		g
GmIDT3	PT	XP_014618511.1	*Glycine max*		g
GmPT01	PT	NP_001335591.1	*Glycine max*		g
GuA6DT	PT	AIT11912.1	*Glycyrrhiza uralensis*		g
GuILDT	PT	AMR58303.1	*Glycyrrhiza uralensis*		g
LaPT1	PT	AER35706.1	*Lupinus albus*	Flavonoid	g
LaPT2	PT	AWK21939.1	*Lupinus albus*		g
LjG6DT	PT	ARV85585.1	*Lotus japonicus*		g
PcM4DT	PT	AYV64464.1	*Psoralea corylifolia*		g
SfFPT	PT	AHA36633.1	*Sophora flavescens*		g
SfG6DT	PT	BAK52291.1	*Sophora flavescens*		g
SfiLDT	PT	BAK52290.1	*Sophora flavescens*		g
SfN8DT-1	PT	BAG12671.1	*Sophora flavescens*		g
CsPT3	PT	DAC76713.1	*Cannabis sativa*		g
CtIDT	PT	AJD80983.1	*Cudrania tricuspidata*		g
MaIDT	PT	AJD80982.1	*Morus alba*		g
HlPT-1	PT	BAJ61049.1	*Humulus lupulus*	Phloroglucinol	h
HlPT2	PT	AJD80255.1	*Humulus lupulus*		h

Ref: Shen G, Luo Y, Yao Y, Meng G, Zhang Y, Wang Y, Xu C, Liu X, Zhang C, Ding G, Pang Y, Zhang H, Guo B. The discovery of a key prenyltransferase gene assisted by a chromosome-level Epimedium pubescens genome. Front Plant Sci. 2022 Nov 14;13:1034943. doi: 10.3389/fpls.2022.1034943. PMID: 36452098; PMCID: PMC9702526.

Table S15. Seeds sequences information used for identification of TPS genes.

#gene_names	NCBI Accession_ID
MsTPSb1	AAC37366.1
MtTPSa1	AAA19216.1
ZmTPSc1	AAA73960.1
GaTPSa2	AAA93065.1
TcTPSd1	AAC49310.1
CmTPSe1	AAB39482.1
AgTPSd6	AAB05407.1
CsTPSf1	AAC49395.1
PfTPSb1	BAA08367.1
PsaTPSc1	AAB58822.1
AgTPSd10	AAB71084.1
AgTPSd5	AAB71085.1
AgTPSd3	AAB70707.1
AgTPSd2	AAB70907.1
SbTPSc1	AAB87091.1
MpTPSa1	AAB95209.1
AgTPSd8	AAC05727.1
AgTPSd9	AAC05728.1
LeTPSa1	AAC39431.1
AgTPSd1	AAC24192.1
SfTPSb1	AAC26016.1
SfTPSb2	AAC26017.1
SfTPSb3	AAC26018.1
EoTPSa1	AAC31570.1
CaTPSa1	CAA06614.1
CmTPSc1	AAD04292.1
SbTPSe1	AAD34294.1
StTPSa1	BAA82092.1
MlTPSb1	AAD50304.1
GaTPSa1	AAD51718.1
LeTPSc1	BAA84918.1
AaTPSa1	AAF61439.1
AgTPSd7	AAF61453.1
AgTPSd11	AAF61454.1
AgTPSd4	AAF61455.1
CsTPSc1	BAA95612.1
AaTPSa3	AAF80333.1
SdTPSc1	BAB03594.1
StTPSb1	AAG01140.1

AtTPSb1	AAG09310.1
lsTPSc1	BAB12440.1
lsTPSe1	BAB12441.1
ZmTPSa1	AAG37841.1
CsTPSe1	BAB19275.1
LhTPSa1	AAG41891.1
PtTPSb1	CAC35696.1
CjTPSa1	AAK54279.1
AaTPSb1	AAK58723.1
QiTPSb1	CAC41012.1
GbTPSd1	AAL09965.1
ArTPSb1	AAL17636.1
AaTPSa2	AAL79181.1
lsTPSa1	AAM11627.1
ClTPSb1	AAM53944.1
ClTPSb3	AAM53945.1
ClTPSb2	AAM53946.1
ClTPSb4	AAM53943.1
StTPSb1-2	AAM89254.1
MaTPSb1	AAL99381.1
CtTPSb1	CAD29734.1
MdTPSb1	AAO22848.1
RcTPSa1	P59287.1
PtTPSd2	AAO61225.1
PtTPSd3	AAO61226.1
PtTPSd4	AAO61227.1
PtTPSd1	AAO61228.1
AtTPSg1	AAO85533.1
AtTPSa1	AAO85539.1
MaTPSa1	AAO85555.1
AmTPSg1	AAO42614.1
AmTPSg2	AAO41726.1
AmTPSg3	AAO41727.1
PsTPSd2	AAP72020.1
TcTPSd2	AAR13860.1
PaTPSd2	AAS47689.1
PaTPSd6	AAS47690.1
PaTPSd7	AAS47691.1
PaTPSd3	AAS47692.1
PaTPSd5	AAS47693.1
PaTPSd4	AAS47694.1
PaTPSd8	AAS47695.1

PaTPSd9	AAS47696.1
PaTPSd1	AAS47697.1
PsTPSd1	ABA86248.1
PgTPSc1	ADB55707.1
PgTPSe1	ADB55708.1
PsiTPSc1	ADB55709.1
PsTPSe1	ADB55710.1
JsTPS	BAJ39816.1

Table S16 Abbreviations and full names correspond in Figure 6.

Gene symbol	Gene description
AACT	Acetyl-CoA *C*-acetyltransferase
HMGS	3-Hydroxy-3-methylglutaryl-CoA synthase
HMGR	3-Hydroxy-3-methylglutary-CoA reductase
MK	MVA kinase
PMK	Phospho-MVA kinase
MPDC	Diphospho-MVA decarboxylase
DXS	1-Deoxy-D-xylulose 5-phosphate synthase
DXR	1-Deoxy-D-xylulose 5-phosphate reductoisomerase
MCT	2-C-methyl-D-erythritol 4-phosphate cytidylyltransferase
CMK	4-(Cytidine 5'-diphospho)-2-C-methyl-D-erythritol kinase
MDS	2-C-methyl-D-erythritol 2,4-cyclodiphosphate synthase
HDS	4-Hydroxy-3-methylbut-2-enyl-diphosphate synthase
HDR	4-Hydroxy-3-methylbut-2-enyl diphosphate reductase
IPPI	Isopentenyl diphosphate Δ-isomerase
GPPS	Geranyl diphosphate synthase
FPPS	Farnesyl diphosphate synthase
GGPPS	Geranylgeranyl diphosphate synthase.

Names are according to the Nomenclature Committee of the International Union of Biochemistry and Molecular Biology (IUBMB)

Other symbol	Description
PEP	Phosphoenolpyruvate
E4P	Erythrose 4-Phosphate
Ac-CoA	acetyl-coenzyme A
HS-CoA	reduced coenzyme A
Pi	orthophosphate
1,3-CHD,5-pentyl	1,3-Cyclohexadiene,5-pentyl
Ppi	pyrophosphate
