# Supplementary material for: Integrating genomic and multiomic data for Angelica sinensis provides insights into the evolution and biosynthesis of pharmaceutically bioactive compounds
Source: Commun Biol. 2023 Nov 24;6:1198. doi: 10.1038/s42003-023-05569-5 (PMC10674023; doi:10.1038/s42003-023-05569-5)
Supplement: Supplementary file 3 — Description of Additional Supplementary Files [file 42003_2023_5569_MOESM3_ESM.pdf]

Description of Additional Supplementary Files

File name: Supplementary Data 1
Description: The 909 genetic variations (affecting 686 genes) were annotated as having a high effect on gene function.

File name: Supplementary Data 2
Description: The detailed information of metabolites identified by metabolite profiling.

File name: Supplementary Data 3
Description: The genes involved in the biosynthesis of coumarins, lignins and lignans.

File name: Supplementary Data 4
Description: The gene numbers of inferred terpenoid backbone biosynthesis pathway (map00900) related genes in A. sinensis (QH), A. sinensis (GS) and other 8 related species annotated using KEGG database.

File name: Supplementary Data 5
Description: Seeds sequences or KEGG annotation information used for gene identification.

File name: Supplementary Data 6
Description: The source data for graphs and charts in the main figures.
